# *Plasmodium* sporozoite excystation involves local breakdown of the oocyst capsule

**DOI:** 10.1038/s41598-023-49442-1

**Published:** 2023-12-14

**Authors:** Sadia Saeed, Annie Z. Tremp, Johannes T. Dessens

**Affiliations:** Department of Infection Biology, Faculty of Infectious and Tropical Diseases, London School of Hygiene & Tropical Medicine, Keppel Street, London, WC1E 7HT UK

**Keywords:** Cell biology, Microbiology

## Abstract

*Plasmodium* oocysts develop on the abluminal side of the mosquito midgut in relatively small numbers. Oocysts possess an extracellular cell wall—the capsule—to protect them from the insect's haemolymph environment. To further maximise transmission, each oocyst generates hundreds of sporozoites through an asexual multiplication step called sporogony. Completion of transmission requires sporozoite egress from the capsule (excystation), but this process remains poorly understood. In this study, we fused the parasite-encoded capsule protein Cap380 with green fluorescent protein in a transgenic *P. berghei* line, allowing live fluorescence imaging of capsules throughout sporogony and sporozoite excystation. The results show that capsules progressively weaken during sporulation ultimately resulting in sporozoite exit through small holes. Prior to formation of the holes, local thinning of the capsule was observed. Our findings support an excystation model based on local, rather than global, weakening of the capsule likely facilitated by local re-orientation of sporozoites and apical secretion.

## Introduction

Malaria remains a major public health burden. In 2021, estimated global malaria cases increased from pre-pandemic levels to 247 million, causing over 600,000 deaths, many among children under five^1^. The disease is caused by infection with apicomplexan parasites of the genus *Plasmodium*, with *P. falciparum* the deadliest among several human malaria parasite species. Malaria control and eradication efforts remain heavily reliant on insecticide-based mosquito control, a strategy that is under threat from concerns over insecticide resistance and environmental impacts. Alternative transmission-blocking measures are therefore urgently needed, including antimalarial drugs that target parasite development in the mosquito^2^.

A neglected part of the *Plasmodium* life cycle involves the oocyst, a life cycle stage that develops on the abluminal side of the mosquito midgut. The oocyst takes centre-stage in an essential parasite multiplication step called sporogony, which generates thousands of sporozoites that infect the vertebrate host after mosquito bite. Malaria parasites suffer severe population losses in the mosquito midgut and only a very small percentage of ookinetes that form in the mosquito midgut lumen successfully transition to oocysts in vivo^3^. During their traversal of the mosquito midgut epithelium, ookinetes elicit strong immune responses in the mosquito host that result in substantial ookinete lysis and/or melanotic encapsulation^4–6^. Ookinete-to-oocyst transformation occurs rapidly after ookinetes reach the abluminal side of the midgut, and oocysts are far more resistant to the mosquito innate immune attack. The widely accepted view is that the oocyst capsule protects the parasite from such immunological responses, and this in turn is thought to be achieved through the incorporation of mosquito components into the capsule structure, masking the oocyst from the insect's innate immune system. Oocysts develop on the midgut epithelium’s basement membrane/basal lamina (BL) and remain attached to this structure throughout their growth and differentiation. Hence, components of the BL such as laminin, collagen and fibronectin constitute prime candidate capsule components. Indeed, laminin has previously been identified in and closely associated with the *Plasmodium* oocyst capsule^7,8^. Furthermore, laminin-coating was shown to inhibit melanotic encapsulation of microbeads injected into the mosquito hemocoel^9^, and laminin knockdown in mosquitoes by RNAi significantly reduced the number of oocysts that successfully developed on the midgut^8^. These findings strongly support the concept that the capsule contains mosquito BL-derived molecules that shield the oocyst from immune effector molecules.

Besides mosquito molecules, the oocyst capsule also naturally contains parasite-encoded proteins: Cap380 (*Plasmodium* oocyst capsule protein 380) was the first protein shown to localise to the *P. berghei* oocyst capsule, and its depletion leads to oocysts that are gradually eliminated^10^. Another protein, Cap93 (*Plasmodium* oocyst capsule protein 93), was shown to localise mostly to the inner face of the capsule and its depletion resulted in markedly fewer and smaller oocysts with a thinner capsule than their wildtype counterparts^11^. Most recently, OSCP (*Plasmodium* ookinete surface and oocyst capsule protein) was reported to co-localise with Cap380 in oocysts, and its genetic depletion resulted in oocysts with a thinner wall that degenerate over time^12^; this very large protein is additionally found on the ookinete surface and affects ookinete locomotion^12^. Importantly, these collective observations are consistent with a protective role for the oocyst capsule. Whilst Cap380 has an endoplasmic reticulum (ER) signal peptide and has the properties of a secreted protein, Cap93 and OSCP each possess a single transmembrane helix and are thus predicted to remain anchored into the oocyst plasma membrane. In this study, we examine the biophysical properties of the capsule and the processes of sporozoite excystation by fluorescently tagging Cap380.

## Results and discussion

### Fusion of Cap380 with GFP allows live fluorescence imaging of the oocyst capsule

**Figure 1:**
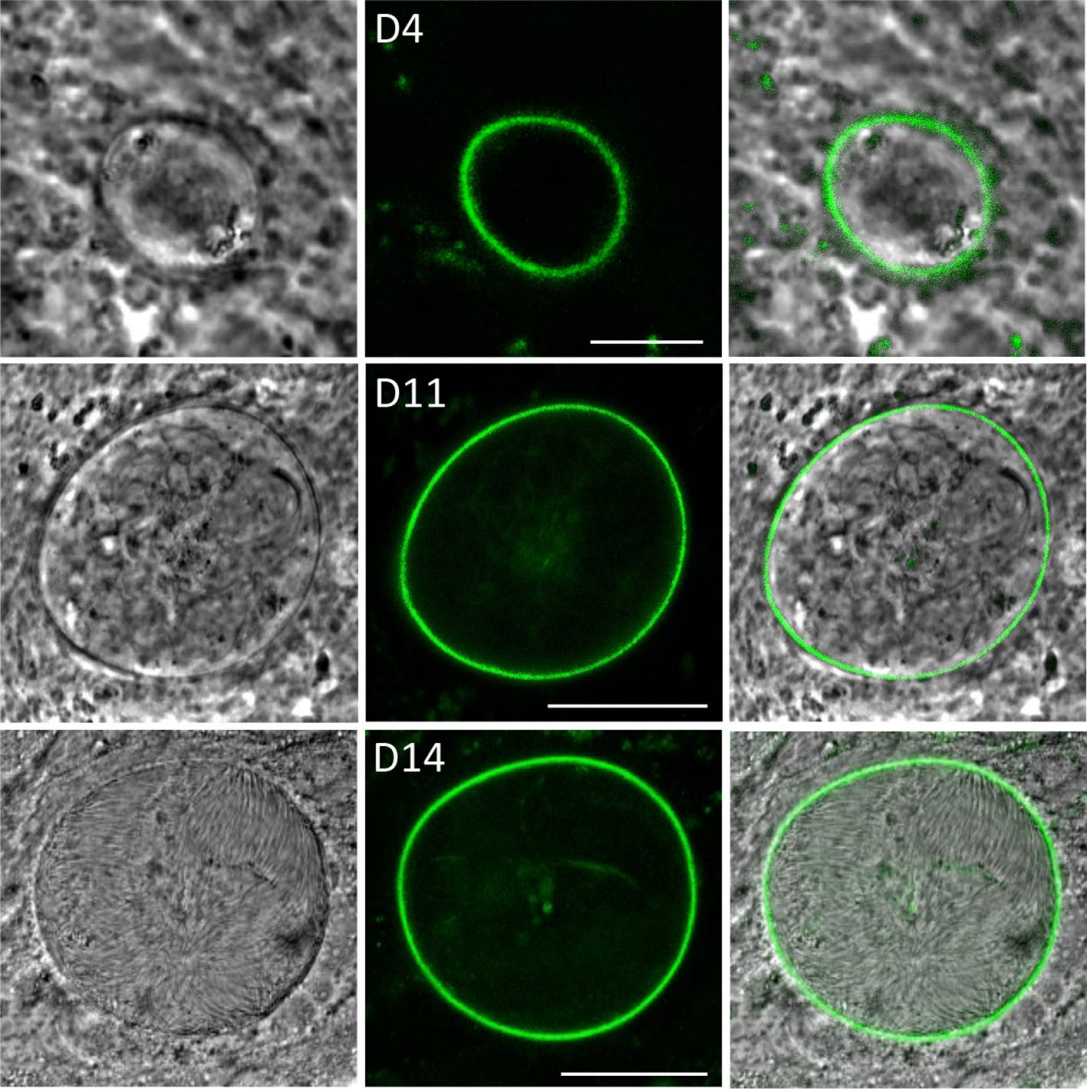
GFP tagging of Cap380 fluorescently labels oocyst capsules. Live confocal GFP fluorescence and phase contrast images of Cap380/GFP-Strep unsporulated oocysts at 4 dpi (D4) and 11 dpi (D11), and a sporulated oocyst at 14 dpi (D14). Scale bar = 5 μm (D4) or 20μm (D11, D14).

To study the oocyst capsule, we set out to generate a parasite line with a fluorescence-labelled capsule structure by fusing the known parasite-encoded capsule protein Cap380^10^ at its carboxy terminus with a dual GFP-Strep tag. As Cap380 is a very large molecule, we chose a gene targeting strategy of single crossover homologous recombination (Supplementary Fig. S1A). Diagnostic PCR across the integration site amplified an approximately 2.4kb fragment of the expected size (Supplementary Fig. S1B), confirming integration of the modified *cap380::gfp-strep* allele into the target locus of the resulting Cap380/GFP-Strep parasite line. Furthermore, the absence of the parental *cap380* allele in clonal populations of Cap380/GFP-Strep was confirmed by diagnostic PCR (Supplementary Fig. S1B). Assessment of GFP expression by confocal fluorescence microscopy of live Cap380/GFP parasites revealed its expression in oocysts from young oocyst through to sporulation (Fig. 1). Fluorescence localised predominantly at the oocyst periphery (Fig. 1), fully consistent with the reported localisation of Cap380 in the capsule using specific antibodies^10^. These observations demonstrate that the capsule can be fluorescently labelled in live oocysts through the fusion of a capsule protein with a fluorescent protein such as GFP.

### The oocyst capsule is flexible and can withstand mechanical pressure

**Figure 2:**
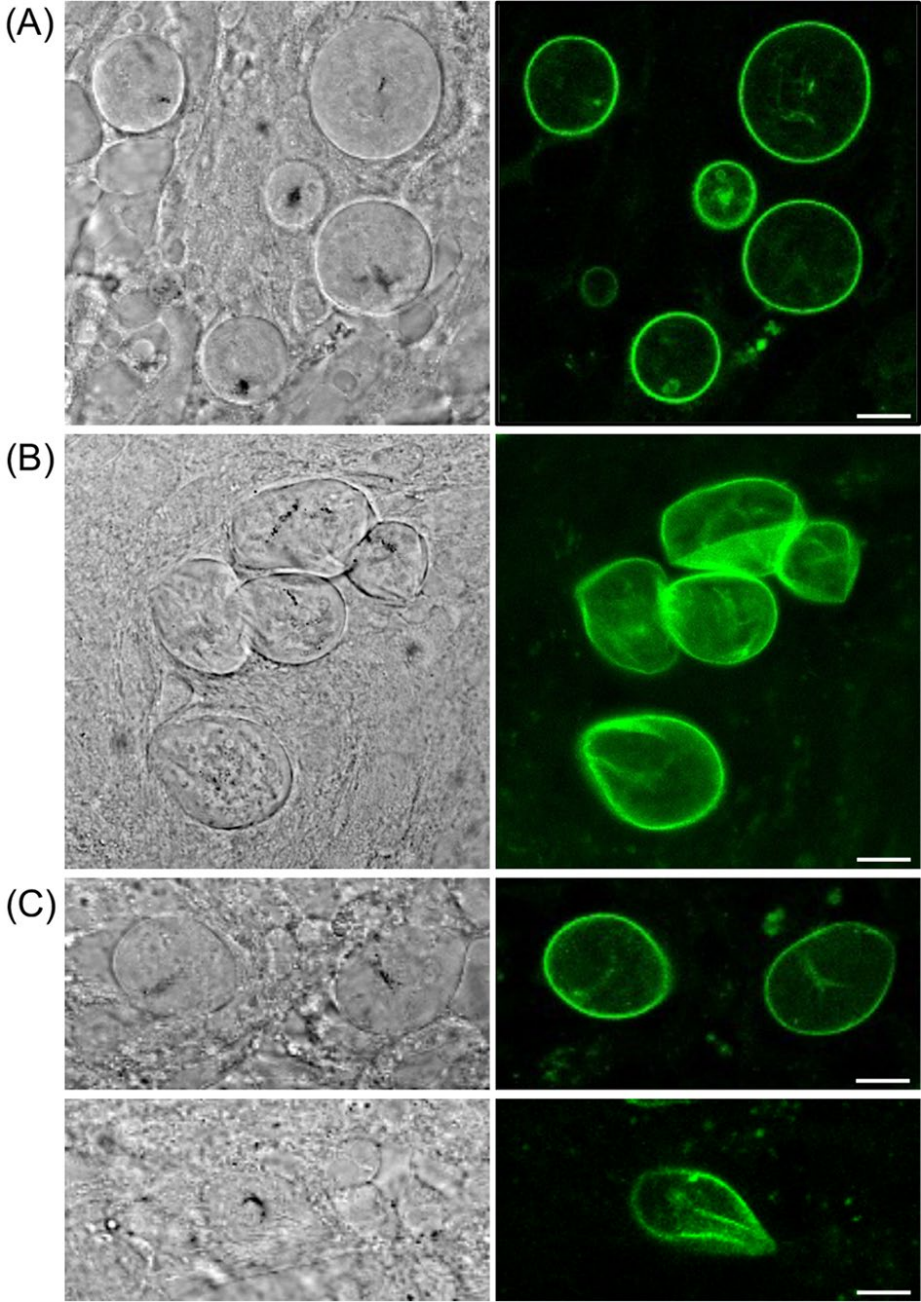
The oocyst capsule is flexible and can withstand mechanical pressure. Live confocal fluorescence and phase contrast images of Cap380/GFP-Strep parasite-infected midguts dissected at 8 dpi in 1 × PBS (**A**), 10 × PBS (B), or 0.1 × PBS (**C**). Lower panels in (**C**) show a ruptured oocyst. Scale bar = 10μm.

The general function of a cell wall is to provide structural support to the cell it envelopes and protect it from mechanical stresses and other adverse environmental factors. While some cell walls are rigid structures (e.g. in coccidian oocysts), others are flexible and obtain rigidity through pressure of the cell within against the extracellular structure (e.g. in plant cells). To investigate the biophysical properties of the *Plasmodium* oocyst capsule, we subjected dissected oocyst-infected midguts to hypo- and hyper-osmotic conditions, followed by live fluorescence imaging (Fig. 2). Hyper-osmotic conditions draw water out of the oocysts, causing their cytoplasm to shrink. This condition resulted in a flattening and folding of the oocyst capsules (Fig. 2B), demonstrating that the structure is not inherently rigid. By contrast, hypo-osmotic conditions cause the oocysts to draw water in. The latter condition caused no discernible change in capsule appearance, but led to a proportion of oocysts bursting (Fig. 2C). Collectively, these observations are consistent with the oocyst capsule being a flexible structure with tensile strength that can withstand a degree of mechanical pressure. By analogy, mechanical pressure on liver schizonts by adjacent, uninfected liver cells leads to rupture of the host cell membrane and subsequent release of merosomes^13^. These biophysical properties of the capsule are well suited to facilitate the rapid growth of the oocyst, which increases up to 1000-fold in volume over the course of sporogony^14^. *Plasmodium* oocysts are not exposed to substantial mechanical stresses and the capsule’s role is more likely to keep out certain environmental components that are detrimental to oocyst development, for example immune effector molecules. Thus, like extracellular matrix, the capsule may acts as a sieve through which water and small polar molecules can easily diffuse, while larger molecules are excluded and require specific mechanisms/transporters for uptake.

### Oocyst capsules progressively weaken during sporulation

**Figure 3:**
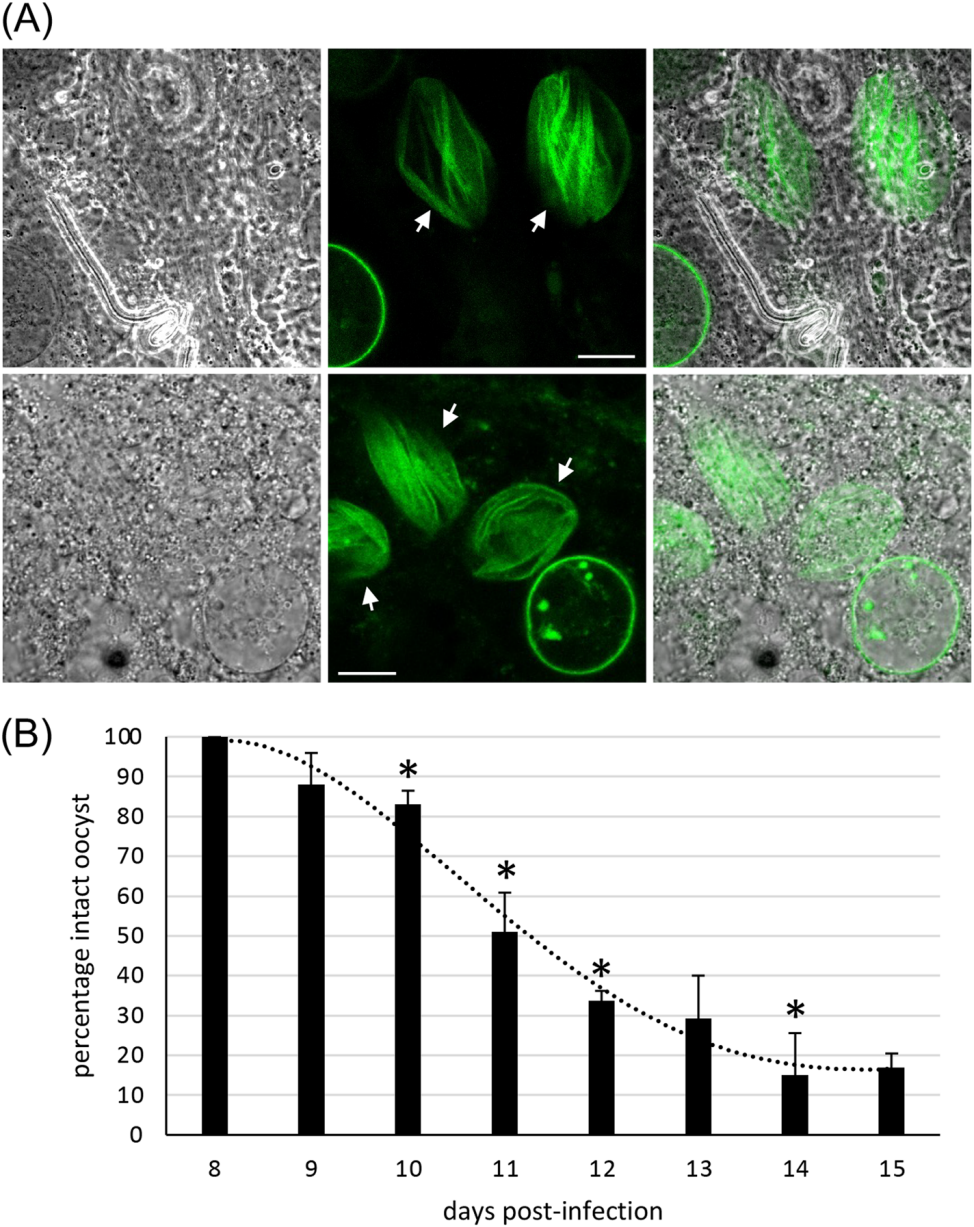
Oocyst capsules progressively weaken during sporulation. (**A**) Live confocal GFP fluorescence and phase contrast images of Cap380/GFP-Strep parasite-infected midguts at 14 dpi. Collapsed capsules (arrows) are visible by fluorescence, but not phase contrast microscopy. Scale bar = 20μm. (**B**) Percentage of intact (spherical) oocysts in oocyst populations at 8 to 15 dpi, with trendline. At least 100 oocysts were scored from at least three independent mosquitoes per time point. Error bars show standard deviation. * depicts significantly different (*p* < 0.05) levels to day before (Student’s t-test).

To study the oocyst capsule during sporozoite egress, we examined Cap380/GFP-Strep oocyst-infected midguts at two weeks post-infected blood feed, when oocyst sporulation is considered to be at its peak^15,16^. In addition to intact oocysts, live fluorescence microscopy revealed the presence of flattened capsules presumably resulting from oocysts vacated, or being vacated, by their sporozoites (Fig. 3A). Notably, these empty capsule structures were not visible when midguts were examined by phase contrast (*i.e.* without capturing GFP fluorescence) (Fig. 3A). This demonstrates that the oocyst does not need to be intact or alive for the capsule to fluoresce, consistent with the capsule constituting a three-dimensional, supramolecular, extracellular matrix that retains water and homeostatic balance.

Given that empty capsules remained visible by fluorescence microscopy (Fig. 3A), we next assessed the proportion of intact, spherical Cap380/GFP-Strep oocysts over the course of sporogony. Compromised (non-spherical) capsules were first observed at 9 days post-infection (dpi) and had reached high levels (> 80%) by 14 dpi (Fig. 3B). We initially assumed this was caused by natural sporozoite excystation. However, we could not rule out that mechanical pressure from the coverslip overlaying the oocyst-infected midguts could be adversely affecting capsule integrity. To test this hypothesis we used ‘Vaseline’ coverslips where a small amount of Vaseline is applied to the bottom side of the coverslip along its rim, which effectively prevents the coverslip from pressing down on the dissected midgut with oocysts. In contrast to conventional coverslips (Fig. 3B), Vaseline coverslips left nearly all (99%) capsules intact at 11 dpi, showing very clearly that mechanical pressure from the coverslip does indeed have a significant effect on capsule integrity. The graph presented in Fig. 3B thus reflects the ability of the oocyst capsules to withstand mechanical pressure from the coverslip, rather than natural excystation, demonstrating that the capsules progressively weaken over time starting around 8 dpi. The unexpectedly high sensitivity of oocysts to coverslip pressure at 11–12 dpi (Fig. 3B) has implications for the assessment of oocyst loads by phase contrast microscopy—an assay routinely used to measure parasite infectivity in the mosquito—as flattened oocysts escape detection (Fig. 3A) resulting in a potential underestimation of oocyst numbers. For this reason oocyst counts should ideally be conducted by 8 dpi and no later than 10 dpi. With the benefit of hindsight, this could for example explain why parasites depleted of oocyst rupture protein 1 (ORP1) or ORP2 give rise to significantly higher oocyst numbers than their wildtype counterparts at 11 dpi^17^. As these mutants are excystation-deficient^17^ it is likely that their oocysts are less sensitive to coverslip pressure.

The graph in Fig. 3B fits the dynamics of sporulation in *P. berghei* oocysts, with sporulation first detected at 8 dpi at very low levels^15^. Hence, it seems plausible that capsule weakening starts with the onset of sporulation, with the graph reflecting the growing subpopulation of sporulating oocysts over time (Fig. 3B). This is further supported by the higher oocyst numbers observed at 11 dpi in *P. berghei* parasites depleted of the LCCL lectin adhesive-like proteins LAP2, LAP4 or LAP6, whose oocysts fail to sporulate^18^, indicating that capsule weakening is not happening, or at least not to the same extent, in the absence of sporozoite formation. Finally, making capsule weakening conditional to sporulation would avoid premature capsule weakening that could result in undesired oocyst loss.

### Examination of fluorescence-labelled oocyst capsules sheds new light on the egress process

**Figure 4:**
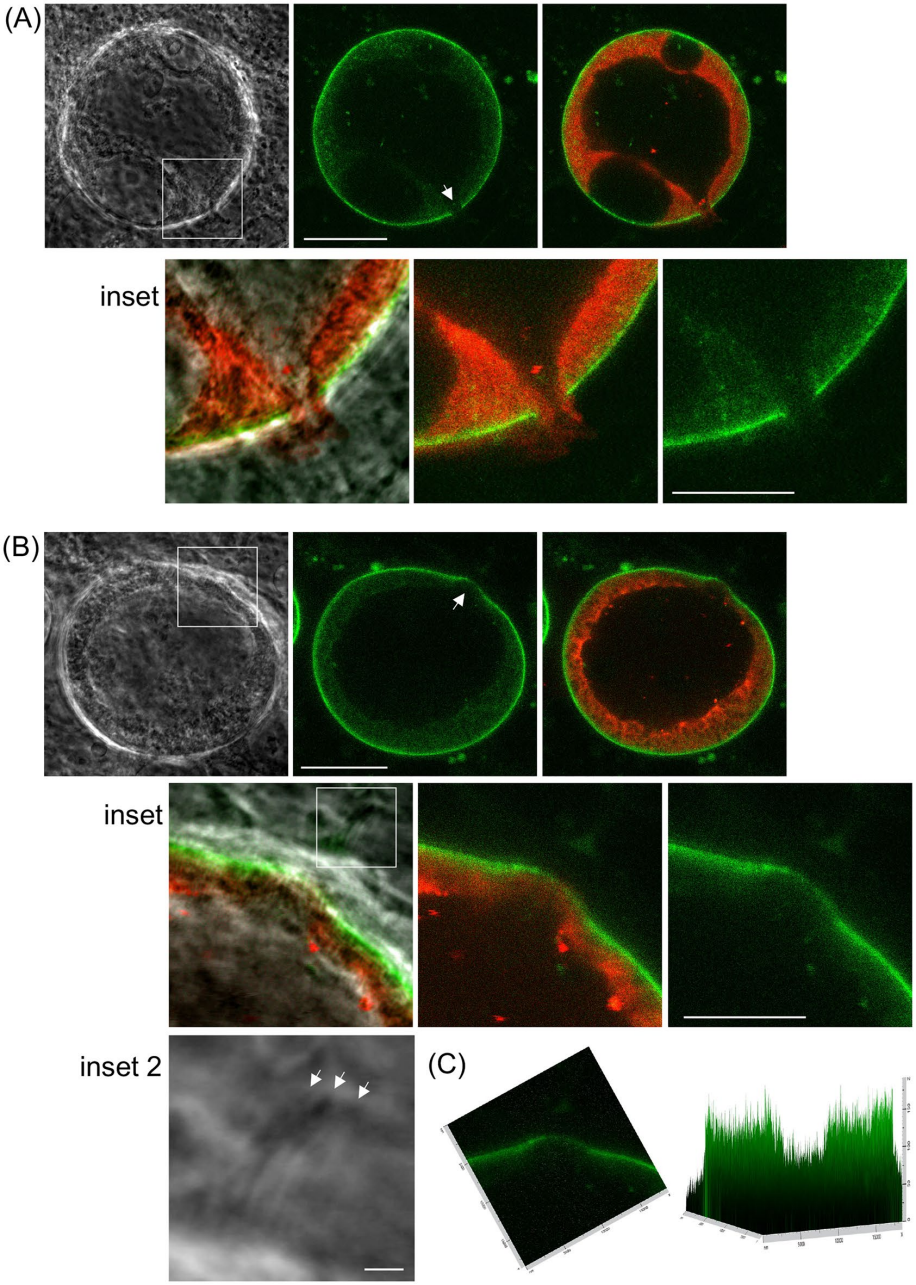
Excystation involves local breakdown of the oocyst capsule. Live confocal fluorescence and phase contrast images of oocysts at 14 dpi of mosquitoes with ookinetes from a Cap380/GFP-Strep x IMC1c/mCherry cross. (**A**) Spherical oocyst with a hole (arrow) in the capsule (green) from which sporozoites (red) are exiting. (**B**) Spherical oocyst with a small bulge (arrow) where the capsule is thinner. Inset 2 shows phase contrast image of sporozoites that appear to have partly traversed the capsule (arrows). (**C**) Graphical representation of GFP fluorescence pixel density (right panel) across the thinned part of the capsule (left panel). Scale bars 20 μm (whole oocysts), 10 μm (inset) and 2 μm (inset 2).

Sporozoites form inside oocysts by budding off the invaginated oocyst plasma membrane (sporoblast) and once fully formed are initially contained within the surrounding capsule^19^. Completion of transmission clearly requires sporozoite egress from this structure, but this process remains poorly understood. In *P. yoelii* oocysts, sporozoites were shown by electron microscopy to emerge through small holes, with perforations getting progressively larger as egress continued^20^. Similar observations were later made for *P. vivax* and *P. falciparum* species^21^. To investigate sporozoite egress in *P. berghei* by fluorescence microscopy, we used our Cap380/GFP-Strep parasite line to visualise the capsule. To also visualise sporozoites, we used our parasite line IMC1c/mCherry that stably expresses the sporozoite-expressed alveolin IMC1c fused to mCherry and displays red fluorescence in mature sporozoites^22,23^. To combine these two fluorescent markers in the same oocyst, the two lines were genetically crossed and the resulting ookinete cultures presented to naïve mosquitoes in membrane feeders, as described^24,25^. Vaseline cover slips were furthermore used to ensure we were not looking at ‘forced’ excystation caused by mechanical pressure from the coverslip on the cells. Using this setup we were able to observe a small number of natural egress events. We found oocysts with red fluorescence on the outside of the capsule near a small opening (Fig. 4A). It is difficult to determine conclusively if oocyst undergoing excystation have one, or multiple, holes in their capsule, as their detection relies on their positioning relative to the focal plane of the microscope objective, and additional holes may be forming at different times. Nonetheless, in the egress events we observed, both using Vaseline and conventional cover slips, we only ever observed a single opening (n = 20) (Supplementary Fig. S2), indicating this is the most common scenario. Egress of merozoites from the red blood cell has also been reported to take place via a single opening in the host plasma membrane and underlying cytoskeleton^26^. It is noticeable that the oocyst shown in Fig. 4A still possesses a substantial sporoblast body, indicating that the oocyst is not yet fully sporulated (*i.e.* has used up all sporoblast for sporozoite formation) and thus implying that sporulation can continue whilst excystation is in progress.

In addition to oocysts with a single opening through which sporozoites were egressing, we observed spherical oocysts containing a small area of their capsule bulging outwards (Fig. 4B). Closer examination in the Z direction showed that in this area the capsule was intact, but markedly thinner (Fig. 4B inset, Fig. 4C). This observation further supports our other data (Fig. 2) that the intact capsule is flexible and pressurized from within, thus causing weaker/thinner parts to be pushed outwards. It seems plausible that this local thinning of the capsule structure precedes the formation of a small opening like the one shown in Fig. 4A. It is noteworthy that what appear to be sporozoites have already partly traversed the thinner part of the capsule (Fig. 4B, inset 2) suggesting that it has become more porous. These collective observations support a model of excystation in which sporozoites facilitate local breakdown of the capsule, possibly through re-alignment in a perpendicular orientation and apical secretion of capsule-degrading enzymes. Apical secretion is dependent on environmental triggers^27,28^, which may be preventing similar secretion by the rest of the sporozoite population. Such a strategy would optimise proteolytic resources and arguably allow faster excystation compared to when the entire capsule is targeted.

It is still not entirely clear what drives sporozoite egress. There is evidence to suggests that excystation is an active process facilitated by sporozoite gliding motility^29^. However, arguing strongly against this concept is the fact that null mutant parasites for thrombospondin-related adhesive protein—whose sporozoites lack productive motility—display normal sporozoite excystation^30^. Our study shows that the intact oocyst capsule achieves its spherical shape by being pressurised, and possesses tensile strength and flexibility to withstand pressure from within by the oocyst (Fig. 2). This opens the possibility that elastic energy stored within the capsule facilitates, at least in part, sporozoite emergence once a hole in the capsule is formed. Indeed, extracellular matrix generally is well-known for its elastic properties^31^. This could explain why in some *P. berghei* oocysts sporozoite egress appears to involve ‘rapid bursting’^29^. Another mode of excystation that was described in *P. berghei* involves groups of sporozoites seemingly captured inside membrane-delineated vesicles (so-called sporosomes by analogy to liver stage merosomes^13^) exiting the capsule, although the potential source of membrane remains obscure^29^. We observed something resembling this in our own experiments (Supplementary Fig. S2). We note that this phenomenon was only observed using conventional coverslips^29^ (Supplemental Fig. S2) and could thus be the result of unnatural pressure on the sporulated oocysts rather than have any biological relevance. Further studies are needed to test this hypothesis.

Our results clearly demonstrate that egress involves the formation of holes in the capsule, but how this is achieved on a molecular level remains poorly understood. Parasites genetically depleted of egress cysteine protease 1 (ECP1, also known as SERA5) are incapable of sporozoite egress and their oocyst capsules are less permeable than equivalent wildtype capsules^16^. These data point to a model in which the oocyst capsule is altered/weakened through a parasite-encoded proteolytic process. Our observation of local thinning of the capsule seemingly preceding sporozoite egress (Fig. 4B,C) argues against a strategy whereby the entire capsule structure is proteolytically weakened resulting in its eventual rupturing at the weakest point. Indeed, this could explain why differences in (the thickness of) capsule structures have not been observed by electron microscopy between wildtype oocysts and those of egress mutants^29^. If proteolytic activity were directed at the entire capsule an overall thinning of the wildtype capsules would be expected. Instead, local thinning supports a model of local weakening of the capsule structure at one or few sites only. We do not currently know if such exit sites are randomly chosen or predetermined. Possible target sites could be micropores that were shown to be present in the oocyst capsule^32^, but further studies are needed to test this hypothesis.

A number of non-enzymatic parasite-encoded molecules have been reported to be involved in sporozoite egress, including circumsporozoite protein^33^, thrombospondin-related protein 1^29^, ORP1 and 2^17^, and *Plasmodium* cysteine repeat modular proteins 3 and 4^34^, indicating that the excystation process is complex and possibly highly orchestrated. Our findings presented here show that oocyst capsule proteins can be successfully tagged without affecting capsule function. This provides a valuable tool for identifying other capsule-associated proteins (e.g. via affinity purification followed by mass spectrometry-based proteomic analysis^35^) to help dissect the molecular processes that underlie excystation in *Plasmodium*.

## Methods

### Ethics statement

As described previously^36^, all laboratory animal work was carried out in accordance with the United Kingdom Animals (Scientific Procedures) Act 1986 implementing European Directive 2010/63 for the protection of animals used for experimental purposes and was approved by the London School of Hygiene & Tropical Medicine Animal Welfare Ethical Review Body and United Kingdom Home Office. Experiments were typically conducted in 6–8 weeks old CD1 mice, specific pathogen free and maintained in filter cages, following ARRIVE guidelines. Animal welfare was assessed daily and upon reaching experimental or clinical endpoints animals were humanely euthanized by exposure to carbon dioxide gas in a rising concentration. Mice were infected with parasites suspended in phosphate buffered saline (PBS) by intraperitoneal injection, or by infected mosquito bite on anaesthetized animals. Intra-erythrocytic parasitemia was monitored regularly by microsampling blood from a superficial tail vein. Drugs were administered by intraperitoneal injection or where possible were supplied in drinking water. Parasitized blood was harvested by cardiac bleed under general anaesthesia without recovery.

### Parasite maintenance, culture and transmission

*P. berghei* ANKA clone 2.34 parasites were maintained as cryopreserved stabilates or by mechanical blood passage and regular mosquito transmission, as previously described^36^. Mosquito infection and transmission assays were as previously described using *Anopheles stephensi*^37,38^ and infected insects were maintained at 20°C at approximately 70% relative humidity under a 12h/12h light/dark cycle. Ookinete cultures were set up overnight from gametocytemic blood as described^39^. For genetic crosses between different lines, equal amounts of blood of comparable gametocytemia were mixed at ookinete culture setup, and following 24h culture blood cells and ookinetes were collected by centrifugation and fed to mosquitoes in membrane feeders.

### Generation and diagnostic PCR of transgenic parasite lines

Plasmid pBS-EGFP-hDHFR^36^ was PCR-amplified with primers GFP-StrepII-F (GGAGGAGGAAGTGGAGGA*GGATCC*GCATGGAGTCATCCTCAATTTGAAAAATAAATTCTAGAAGATCCCGTTTTTCTTACTTATATA) and GFP-StrepII-R (CTCCACTTCCTCCTCCTTTGTATAGTTCATCCATGCCATGTGTAAT), followed by circularization of the PCR product by In-Fusion to give pBS-GFP-Strep. The 3’-proximal 2.2kb of the CAP380 coding sequence was PCR amplified with primers CAP380-F (TTGGGCTGCAGTCGATTCGAATGTGAATCCAGGG) and CAP380-R (ATGAGGGCCCCTAAGCTTGCTCTTCGCAACACATTTAG) and cloned into SalI/HindIII-digested pBS-GFP-Strep by in-fusion cloning to give plasmid pBS-Cap380/GFP-Strep. Plasmid pBS-Cap380/GFP-Strep was linearized with *Bgl*II prior to transfection of purified schizonts to generate parasite line Cap380/GFP-Strep by single crossover homologous recombination. Parasite transfection, pyrimethamine selection and limiting dilution cloning were performed as described^40,41^. Primers P1 (GTGTTTTGCGCGTCGG) and P2 (GTGCCCATTAACATCACC) were used to PCR across the 5′-integration site, amplifying an approximately 2.4 kb fragment. The absence of the unmodified *cap380* allele was confirmed in clonal lines of Cap380/GFP-Strep by PCR with primers P1 and P3 (CATGCACTGCAATGCCAC), which amplifies an approximately 2.3kb fragment from parental parasites only.

### Microscopy

Live parasite samples were assessed, and images captured, on a Zeiss LSM880 laser scanning confocal microscope using 100 × or 40 × oil objectives and ZEN Blue version 3.0 software, as described^36^. Protein expression and subcellular localisation were assessed and compared in oocysts from mosquito batches infected with uncloned and cloned populations of transgenic parasites to ensure results were consistent and representative.

### Supplementary Information

Supplementary Figures.

## Data Availability

All data generated or analysed during this study are included in this published article and its Supplementary Information files.
