# Supplementary material for: Plasmodium sporozoite excystation involves local breakdown of the oocyst capsule
Source: Sci Rep. 2023 Dec 14;13:22222. doi: 10.1038/s41598-023-49442-1 (PMC10721906; doi:10.1038/s41598-023-49442-1)
Supplement: Supplementary file 1 — Supplementary Figures. [file 41598_2023_49442_MOESM1_ESM.pdf]

Plasmodium sporozoite excystation involves local breakdown of the oocyst capsule

Sadia Saeed, Annie Z. Tremp, Johannes. T Dessens

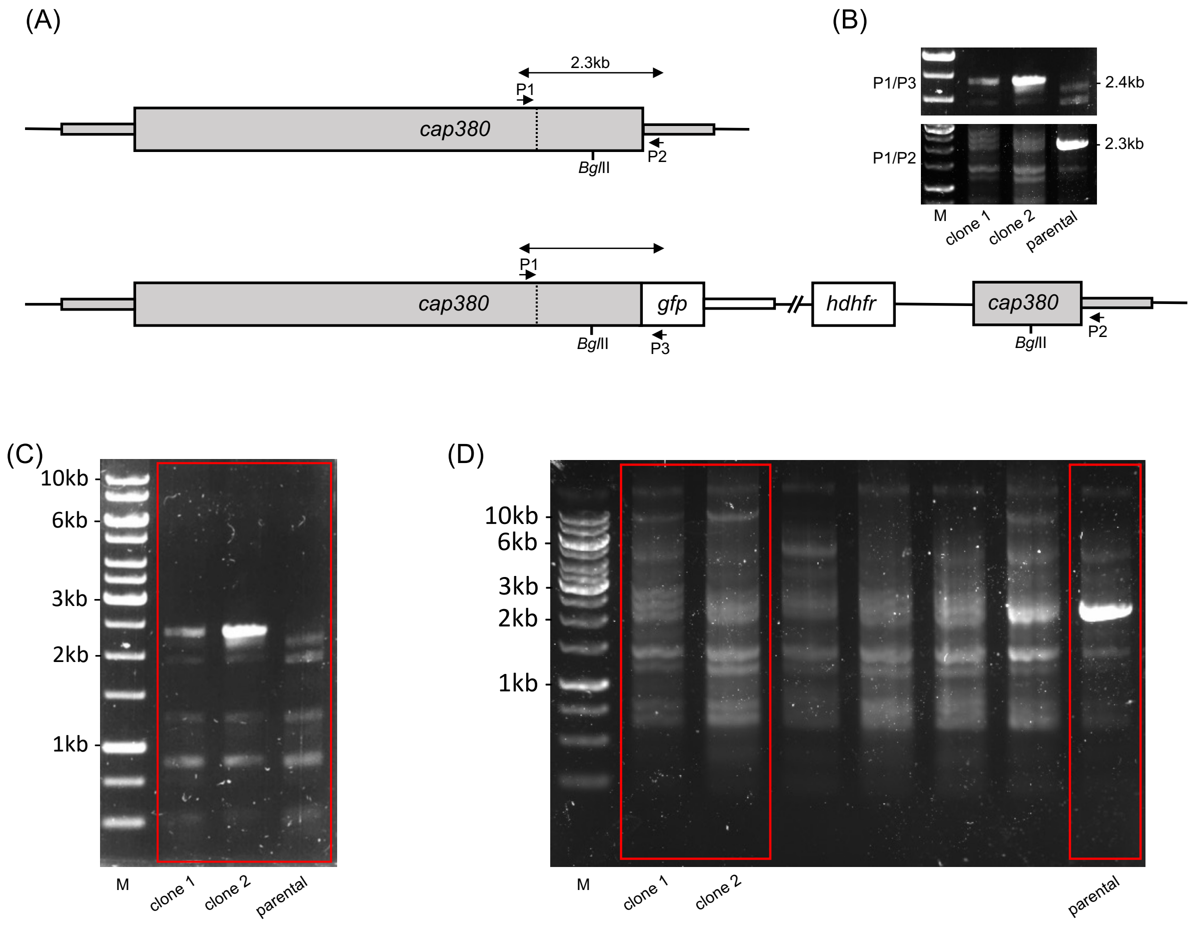

Figure S1. Generation and genotyping of *P. berghei* parasite line Cap380/GFP. (A): Schematic diagram of the modified *cap380* allele in parasite line Cap380/GFP generated by single crossover homologous recombination. The *cap380* gene is shown in grey with coding sequence (wide bars) and 5′ and 3′ untranslated regions (narrow bars). Also shown are the *Bgl*II restriction site used for single crossover homologous recombination, the *gfp* module, the selectable marker (*hdhfr*), and positions of primers P1-P3 used for diagnostic PCR amplification. (B): PCR with primers P1 and P3 diagnostic for integration of the modified *cap380::gfp* alleles into the *cap380* locus, or with primers P1 and P3 diagnostic for absence of the unmodified parental *cap380* allele. See Materials section for primer sequences. (C): Uncropped DNA agarose gel corresponding to top panel in (B). (D): Uncropped DNA agarose gel corresponding to bottom panel in (B). Lanes used in (B) are marked by red boxes in (C) and (D), and corresponding samples are named underneath. M denotes lane with DNA ladder, with sizes shown on left-hand side in (C) and (D).

***Plasmodium* sporozoite excystation involves local breakdown of the oocyst capsule**

Sadia Saeed, Annie Z. Tremp, Johannes. T Dessens

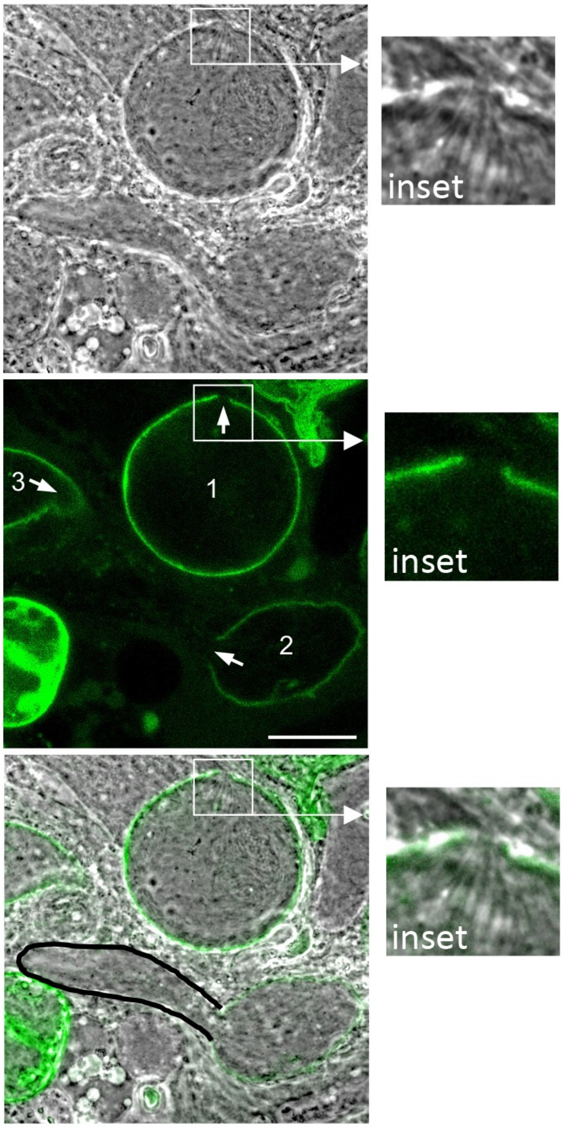

Figure S2. Sporozoite excystation observed using conventional coverslips. Live confocal GFP fluorescence and phase contrast images of Cap380/GFP parasite-infected midguts at 14 dpi, showing three oocysts (1-3) with holes (arrows) in their capsules. Oocyst 1 is spherical and shows sporozoites egressing through the hole (shown at higher magnification in the insets). Oocyst 2 shows a ‘blob’ of oocyst content outside of the capsule (delineated by black line in bottom panel) that is continuous and uniform with the internal oocyst and resembles a sporosome. Scale bar 20μm.
